# Electromass Transfer in the System “Cation Exchange Membrane—Ammonium Nitrate Solution”

**DOI:** 10.3390/membranes12111144

**Published:** 2022-11-15

**Authors:** Olga Kozaderova, Oleg Kozaderov, Sabukhi Niftaliev

**Affiliations:** 1 Faculty of Ecology and Chemical Technology, Voronezh State University of Engineering Technologies, 394036 Voronezh, Russia; 2 Faculty of Chemistry, Voronezh State University, 394018 Voronezh, Russia

**Keywords:** electrodialysis, electromass transfer, ammonium nitrate, cation exchange membrane

## Abstract

The paper describes an experimental study and the mathematical simulation of the electromembrane transfer of cations of weak electrolytes (namely, ammonium ions), hindered by hydrolysis reactions taking place in the surface layers of the cation exchange membrane. Using the finite element method, we found a solution to the corresponding diffusion-kinetic electrodialysis problem in potentiostatic mode. Based on the experimental data and the results of theoretical simulation, we analyzed the effect of hydrolysis on the concentration polarization of the electromembrane system and the transport characteristics of ions, and suggested a mechanism of transfer of the components of the ammonium nitrate solution through the cation exchange membrane.

## 1. Introduction

Electrodialysis is an electromembrane process which is widely used in the food and chemical industries, as well as for desalination of sea water and production of ultrapure water [1,2,3]. Electrodialysis is often involved in the processing of carbonate, phosphate, and ammonium-containing solutions, including waste products of metal phosphate coating [4], industrial wastewater [5,6], and amino acid solutions containing ammonium sulphate and monosodium phosphate [7,8,9]. However, all these technological applications of electrodialysis are hindered by the need to use weak electrolytes. Concentration/desalination during electromembrane processing of such aqueous solutions is more complicated as compared to the salt solutions of strong acids and bases because the system contains products of interaction of weak electrolytes (carbonate, phosphate, and ammonium ions) with the solvent. The electrodialysis process can be hindered at various stages of polarisation, both at under-limiting and overlimiting currents. This results in a decrease in the extraction of components from the solution due to the fact that they form weak electrolytes following the changes in the pH in the desalination compartment [5]. The amount of energy required for the migration through membranes also increases [6], and the effectiveness of electrodialysis depends to a large extent on the buffering capacity. Therefore, it is important to develop a scientific basis for the transfer of ions through cation and anion exchange membranes during the electrodialysis of weak electrolyte solutions.

**Figure 1:**
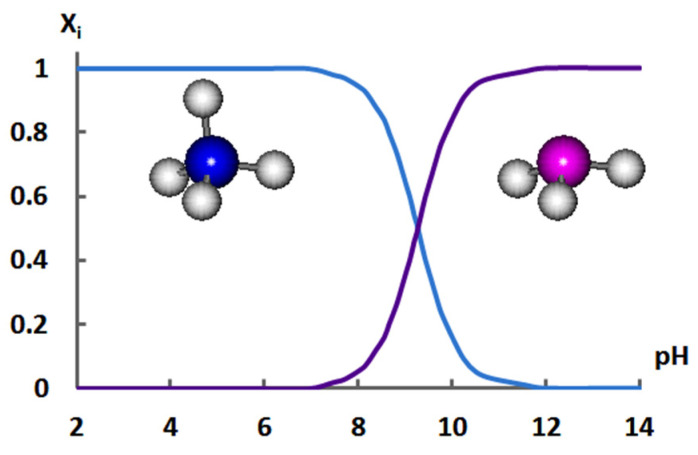
The influence of the pH on the distribution of ammonium/ammonia ion and molecular forms in water at the temperature of 25 °C.

The existing studies focus mostly on the electromass transfer of carbonates/hydrocarbonates, phosphates [10,11,12,13], and amino acids [14,15,16,17] during electrodialysis. Ammonium-containing aqueous solutions have not been thoroughly studied yet. Such solutions demonstrate characteristic behavior in electromembrane systems resulting from the dependence of the ratio of ion and molecular forms of ammonium/ammonia on the pH (Figure 1).

The following reactions take place in solutions of ammonium salts characterized by the corresponding hydrolysis *K_h_* and dissociation *K_d_* constants [18]:

NH_4_^+^ + H_2_O ↔ H_3_O^+^ + NH_3_, *K*_h_ = 5.6·10^−10^, (1)

NH_3_·H_2_O ↔ NH_4_^+^ + OH^−^, *K*_d_ = 1.8·10^−5^. (2)

The presence of the H^+^ and OH^−^ ions in the Equilibria (1) and (2) determines the effect of the changes in the pH in the near-membrane layers of the solution, as well as the local changes in the pH in proximity to the surface of heterogeneous membranes taking place during electrodialysis, on the ammonium/ammonia ratio, whose variations contribute to the effectiveness of the transfer of ions inside, and through the membranes during electrodialysis of aqueous solutions containing ammonium.

Experimental studies of membrane processes (including electrodialysis) in ammonium-containing solutions are described in [19,20,21,22,23,24,25,26,27,28,29,30,31]. Ref. [19] focused on electrodialysis of solutions obtained in the process of production of ceric ammonium nitrate. Refs. [20,21,22] considered the possibility of using electrodialysis as one of the techniques during the processing of nitrogen-containing wastewater, aiming at reusing the water. Ref. [23] studied electrodialysis desalination of secondary steam condensate formed in the process of production of ammonium nitrate using ion exchange membranes, including profiled membranes, in laboratory, and experimental—industrial electrodialysis stacks. Ref. [24] considered the selectivity of nanofiltration membranes to ammonium and nitrate ions in the presence of sodium chloride. Ref. [25] demonstrated the effectiveness of bipolar electrodialysis in obtaining HCl and NH_3_·H_2_O and desalination of ammonium chloride containing wastewater. In some cases, conversion of ammonium nitrate may be more preferable than traditional methods of processing of such solutions [26] because it results in products which can be used in the same technological process. In [28], the dependence of ammonium fluxes through a cation exchange membrane as a function of current density was studied. The detected increase in ammonium fluxes when the limiting current is exceeded is explained by conjugated transport with hydrogen ions. In [29], the treatment of solutions using bipolar electrodialysis technology was considered. The review in [30] analyzed research, technologies, mechanisms, economic feasibility, and prospects for the use of electrodialysis extraction of ammonium from wastewater. Ref. [31] demonstrated that the outlet flux decreases for an anode membrane following an increase in the current density during electromembrane separation of ammonium nitrate solution. This is caused by a change in the pH of the acidified anode permeate. On the contrary, the outlet flux increases for the cathode membrane, which occurs due to the change in the pH of alkalized cathode permeate.

However, very few studies provide a theoretical substantiation for the transfer of ammonium ions through membranes [32,33,34]. At the same time, theoretical simulation is widely used in the field of electrochemistry of membrane processes, including the processes of electrochemical separation by means of charged ion-exchange membranes. The modelling process is hindered by the introduction of several subsequent stages in the membrane separation process: transport of the electrolyte components (ions, neutral molecules, and solvent molecules) towards the solution/membrane interface; transfer of the components of the system through the membrane; removal of the splitting products into the external solution on the other side of the membrane; hydrolysis and dissociation reactions (for weak electrolytes). Refs. [33,34] suggested and substantiated the mechanism of transfer of ammonium in the form of co-ions through the anion exchange membrane. Ref. [32] presented the results of experimental and theoretical studies of the processes involving ammonium ions, which take place in the membrane and the compartments of a microbial electrolysis cell consisting of anodic and cathodic compartments separated by a cation exchange membrane. It demonstrated that the electromass transfer of the system’s components is significantly affected by the products of electrochemical reactions due to variations in the pH in electrode sections.

In this paper, we considered the processes occurring in cation exchange membranes involving the components of an aqueous ammonium nitrate solution during its electrodialysis in an electrodialysis concentrator. The article focuses on the transfer of ammonium as a counterion and the effect of the pH, which is not as predictable as the effect of acids or alkali in membrane electrolysis, especially at under-limiting currents. The theoretical model of the electrodialysis process presented here is based on the Nernst–Planck equation combined with additional ratios and boundary conditions, which determine the physicochemical properties of the membrane, the transfer of ions in the external solution, and the chemical reactions (dissociation, hydrolysis) in the solution and the membrane.

Earlier [27], we studied the electrodialysis of an ammonium nitrate solution (0.012 mol/dm^3^) in the range of current density (*i*) exceeding the limiting density (*i*_lim_): 1 < *i*/*i*_lim_ < 6. An analysis of the dependence of NH_4_^+^ ion flux on the current density demonstrated that the flux density first increases and then decreases when the current density approaches the limiting value. If the current density is increased further, the flux of NH_4_^+^ counterions through the membrane increases again. We explained this dependence in terms of “barrier effect” and “facilitated migration” [16,35]. The flux of ammonium ions decreases due to their interaction with hydroxyl ions generated in the purifying compartment during heterolytic water splitting, when the limiting current density is exceeded on the cation exchange membrane/solution surface. As a result of further increase in the current density, hydrogen ions move towards the surface of the cation exchange membrane. These ions are formed during heterolytic dissociation of water on the boundary with the anion exchange membrane. The pH of the depleted layer of the solution decreases in the proximity to the cation exchange membrane. The concentration of NH_4_^+^ in the solution increases, which leads to an increase in their flow. This is what we refer to as the facilitated migration effect.

It is obvious that the transfer of ammonium through the cation exchange membrane at under-limiting current has its specific features. Thus [36,37] demonstrated that the pH inside the anion exchange membrane is higher than the pH of the equilibrium solution. It is caused by the Donnan exclusion of H^+^ ions as co-ions from the anion exchanger. It is possible that the same is true for the cation exchange membrane as well. This is why it is necessary to consider the possibility of participation of ammonia in the protonation/deprotonation reactions when describing the transfer of ammonium through the cation exchange membrane, even at under-limiting current.

The purpose of our study was to investigate the characteristic patterns of the transfer of cations of weak bases through cation exchange membranes during electrodialysis at under-limiting current based on the analysis of experimental data and theoretical simulation.

## 2. Materials and Methods

### 2.1. Ion Exchange Membranes and Aqueous Solutions 

Electrodialysis was performed using heterogeneous ion-exchange membranes MK-40 and MA-41. The electrical conductivity was measured for cation-exchange membranes MK-40 and MK-41 (Shchekinoazot, Russia [38]) (Table 1). MK-40 is a sulphonic acid cation exchange membrane; MA-41 is an anion exchange membrane with quaternary ammonium fixed groups; MK-41 is a cation exchange membrane with phosphonic acid groups characterized by various degrees of dissociation (pK_1_ = 2.1; pK_2_ = 6.7). The change in the color of the pH-indicator inside sulphonic cation exchangers in NH_4_^+^ or K^+^ ionic form was visualized using MK-40 and a KU-2–8 granular sulphonic cation exchanger, which was used to produce the MK-40 membrane.

To determine the characteristic features of the transfer of ammonium cations in the studied electromembrane systems, we conducted experiments with ammonium and potassium salts. In our experiments, we used nitrate solutions with a concentration of 0.01 mol∙dm^−3^. Potassium and ammonium ions demonstrate similar characteristics in aqueous solutions. Table 2 presents the information regarding the radii of these ions and their diffusion coefficients, together with the corresponding characteristics of other ions and molecules considered in the article.

The concentration of ammonium and potassium ions in the solution was determined by means of direct potentiometry [41,42]. NH_4_^+^ and K^+^ selective electrodes with an ion-sensitive membrane made of polyvinyl chloride film were used. Their potential was measured in the analyzed solution with regard to a silver chloride reference electrode. Direct potentiometry was also performed to determine the pH of the solutions using a galvanic cell consisting of a glass indicator electrode ESP-01-14/7 and a silver chloride reference electrode. To calculate the ion fluxes, we determined the concentration of hydrogen ions in the concentrate compartment of the electrodialysis cell using acid–base titration.

### 2.2. Assessment of the Ion Composition of the Cation Exchange Membrane in Strong and Weak Electrolyte Solutions

To determine the pH of the internal solution of the cation exchange membrane, we used the method described in [36]. A 1.5 × 1.5 cm^2^ sample of the membrane in a NH_4_^+^ or K^+^ ion form was put into distilled water (30 cm^3^). The pH of the equilibrium solution in contact with the membrane and the concentration of NH_4_^+^ or K^+^ cations (Cat^+^) in the solution were determined by means of the potentiometric method. The concentration of ions in the membrane (or the internal solution of the membrane) ${\bar{c}}_{i}$ was calculated using the Nikolsky equation

(3) $$\frac{{\bar{c}}_{H^{+}}}{{\bar{c}}_{{\mathrm{Cat}}^{+}}}=\tilde{K}\frac{c_{H^{+}}}{c_{{\mathrm{Cat}}^{+}}}.$$

Here $\tilde{K}$ is the equilibrium constant of ion exchange between the membrane and the solution. According to [36], it is assumed to be equal 1. Moreover, the conditions of the local electrical neutrality on the membrane/external solution interface were taken into account:

(4) $$\sum_{i}z_{i}c_{i}=0,$$

(5) $$\sum_{i}z_{i}{\bar{c}}_{i}=\bar{Q}.$$

Here, *c*_i_ and ${\bar{c}}_{i}$ are the concentrations of the ions of the i-th type in the solution and membrane respectively (i = K^+^ in KNO_3_ solution or NH_4_^+^ in NH_4_NO_3_ solution, NO_3_^−^, H^+^, OH^−^), z*_i_* is its charge number, and $\bar{Q}$ is the total exchange capacity of the membrane.

**Figure 2:**
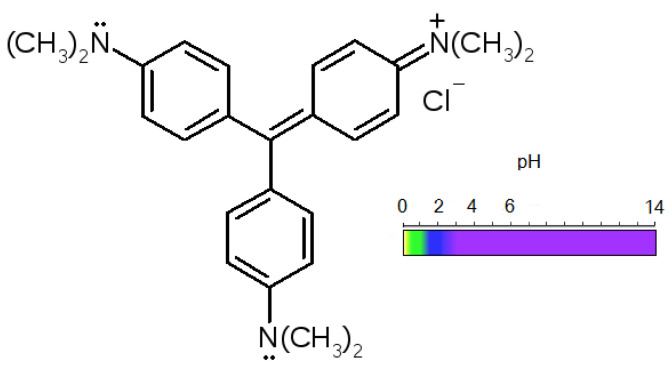
The structural formula of methyl violet and changes in the color of the indicator depending on the pH of the solution.

The pH of the internal solution of the sulphonic cation exchange membrane in various ionic forms was additionally determined using a 0.1% (wt.) methyl violet aqueous solution (Figure 2).

The color of methyl violet changes from greenish-yellow to blue when the pH is 0.5–2.0; when the pH is 2–3 the color changes from blue to violet. The change in the color of the methyl violet indicator in the acidic solution is caused by the successive addition of protons to its groups:

(6) $$-\ddot{N}{\left(CH_{3}\right)}_{2}+H^{+}\overset{}{\to }-N^{+}(H){\left(CH_{3}\right)}_{2}.$$

The changes in the color of the indicator in the studied ion exchange membranes were visualized using a Levenhuk 625 optical microscope with a M1400 Plus camera. Samples of the MK-40 membrane and the KU-2-8 granular ion exchanger in NH_4_^+^ and K^+^ forms were put into water that contained methyl violet for 24 h. Next, the membranes and granules were separated from the solution, dried with filter paper, and studied under the microscope.

### 2.3. The Electrical Conductivity of Ion Exchange Membranes

The electrical resistivity of ion exchange membranes was measured using the contact-difference method [43]. The true resistivity of the sample of the studied membrane ($\bar{R}$, Ohm) was calculated based on the difference between the resistivity of a pair of membranes and the resistivity of a single membrane, which allowed us to avoid the influence of the membrane/electrode interfaces. The values of the true electrical resistivity of the membrane were used to determine its specific electrical conductivity (*k*_m_, Ohm^−1^·cm^−1^), based on its thickness *d* (cm) and the area of the electrodes *A* (cm^2^):

(7) $$k_{m}=\frac{d}{\bar{R}A}.$$

**Figure 3:**
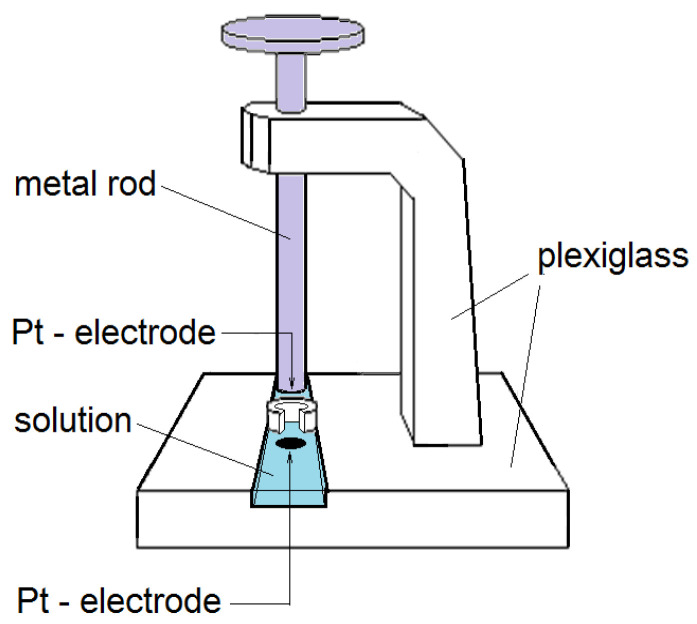
A cell for measuring the electrical resistivity of the membranes using the contact-difference method.

All the measurements were conducted in the cell whose scheme is shown in Figure 3. Electrodes were located in a vessel filled with either water or a salt solution with a set concentration. The measurements were conducted at 100 kHz using a Tesla BM-507 impedance meter.

### 2.4. Electrodialysis of Aqueous Solutions of Ammonium and Potassium Nitrate 

**Figure 4:**
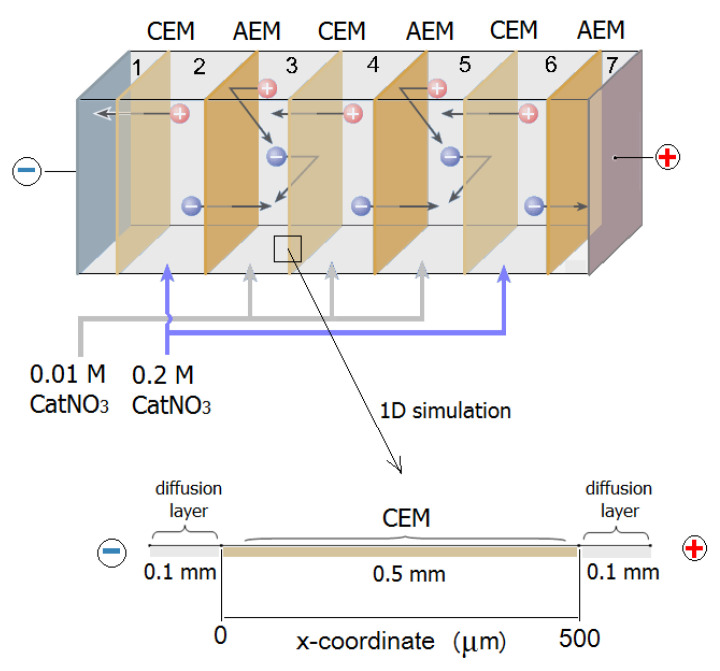
A scheme of transfer of ions during electrodialysis of aqueous solutions of potassium and ammonium nitrates. AEM—anion exchange membranes, CEM—cation exchange membranes, 1–7—compartments.

Electrodialysis of ammonium nitrate and potassium nitrate solutions was performed in a cell demonstrated in Figure 4 using selective polarization of ion exchange membranes [27].

**Figure 5:**
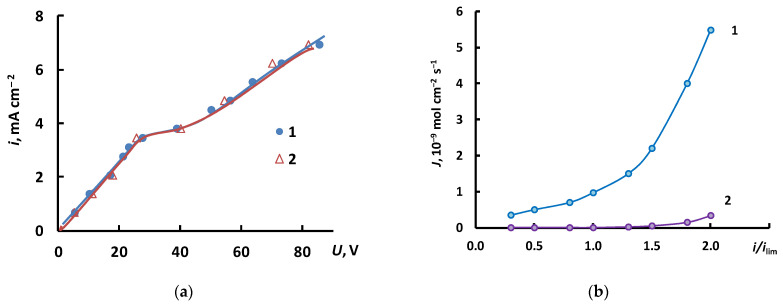
(**a**)—Volt-ampere curves of the electrodialysis cell; (**b**)—Dependence of H^+^ ion flux through the MK-40 cation exchange membrane on the dimensionless current density during electrodialysis; 1—NH_4_NO_3_, 2—KNO_3_.

The process was carried out in a galvanostatic mode, setting the operating value of the current density. After the stationary state was established in the system, solutions were sampled for analysis and voltmeter readings were taken to construct a volt-ampere characteristic. The flow rate of the solution was 5 cm^3^/min, the solution temperature was 25 °C, the active area of one membrane was 14 cm^2^, and the intermembrane distance was 0.1 cm. The value of the limiting current in the system was estimated by the volt-ampere characteristics of the cell. Figure 5a shows the general volt-ampere curves of the electrodialysis cell. It can be seen that the value of the limiting current density is 3.5 mA/cm^2^.

The purpose of our experiment was to compare the fluxes of H^+^ ions through the cation exchange membrane from the desalination compartment to the concentrate compartment in solutions of ammonium and potassium salts. A specific feature of selective polarization of ion exchange membranes is that NH_4_NO_3_ solutions of various concentrations were allowed to flow through the studied desalination compartment 4 and desalination compartments 2 and 6 (the analyzed solution with the concentration of 0.01 mol⋅dm^−3^ flew into compartment 4, and a 0.2 mol⋅dm^−3^ solution was poured into compartments 2 and 6). As a result, the limiting current density was reached on the cation exchange membrane between compartments 3 and 4, and on the anion exchange membrane between compartments 4 and 5. According to the Levich equation [44], the limiting current density *i*_lim_ is proportional to the concentration of the processed solution. The calculation of *i*_lim_ for compartments 2, 4, and 6 in the electrormembrane system demonstrates that it is possible to select a current density range where *i*_lim_ cannot be reached on any anion exchange membrane between compartments 2 and 3, or any cation exchange membrane between compartments 5 and 6. Then, by determining the change in the concentration of H^+^ ions in the solution in compartment 3, we can calculate the fluxes from compartment 4 through the cation exchange membrane using the formula:

(8) $$J_{i}=\frac{\Delta c_{i}V}{S\tau }.$$

Here *J*_i_ is the ion flux, mol/(cm^2^·s); Δ*c*_i_ is the change in the concentration of ions in the compartment, mol/dm^3^; *V* is the sample volume of the solution, dm^3^; *S* is the area of the surface of the membrane, cm^2^; *τ* is the time of sampling, s. The sampling was performed under steady-state conditions.

## 3. Results

Figure 5b shows H^+^ ion fluxes through the MK-40 cation exchange membrane during the electrodialysis of ammonium nitrate and potassium nitrate represented as a function of the dimensionless current density.

**Figure 6:**
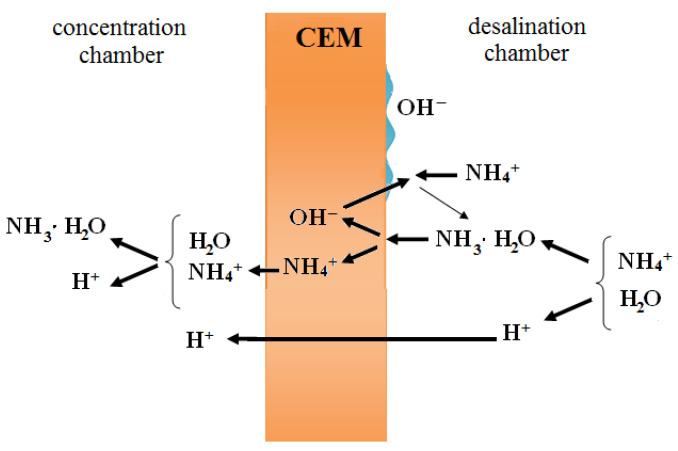
The transfer of ions through the cation exchange membrane: hydrolysis effect.

As we can see, H^+^ ion flux in ammonium nitrate, as opposed to KNO_3_, is not negligibly small even at under-limiting current. Measurements of the pH of the solution in the concentrate compartments demonstrated that, when ammonium nitrate is used, the solution is acidified at *i*/*i*_lim_ < 1. This is not characteristic of electrodialysis of salts containing cations of a strong electrolyte. This fact allowed us to use a particular approach to account for the behavior of ammonium ions in the studied system. The approach was developed in [11,12] to describe the transport of anions of salts of weak acids, and it takes into account the hydrolysis reaction taking place in the external diffusion boundary layer and the internal porous solution of the membrane. NH_3_⋅H_2_O particles formed during the hydrolysis enter the membrane on the depleted interface and transform into NH_4_^+^ ions, because the pH of the internal solution of the membrane is lower than the pH of the external solution. Due to the Donnan exclusion and the effect of direct current, OH^−^ ions return to the depleted solution, where they react with NH_4_^+^ ions, which results in an increase in the concentration of NH_3_⋅H_2_O near the cation exchange membrane. Ammonium cations formed in the internal solution of the membrane migrate towards the opposite boundary of the membrane and transfer to the enriched solution, where they interacted with water molecules, thus causing a decrease in the pH of the solution in the concentrate compartment. Thus, the pH of the depleted solution increases, while the pH of the enriched solution, on the contrary, decreases, which can be viewed as the transfer of H^+^ through the cation exchange membrane (Figure 6).

**Figure 7:**
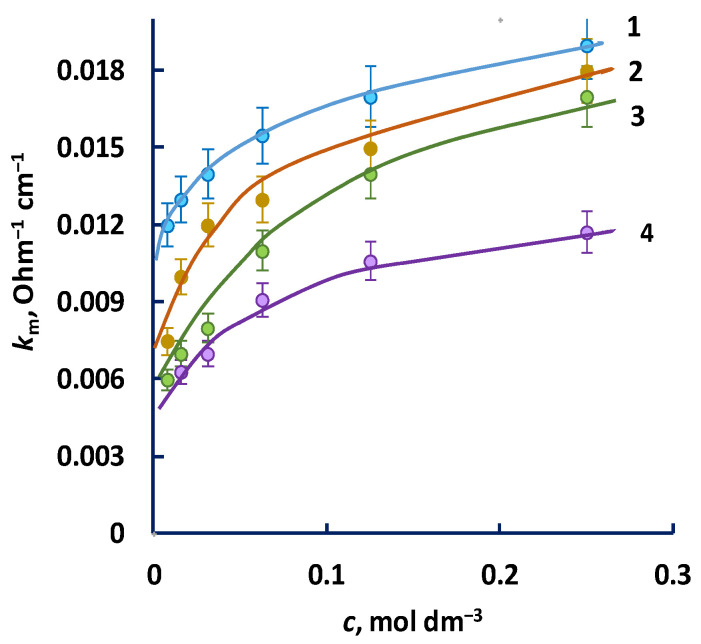
Electrical conductivity of ion-exchange membranes (*k_m_*) represented as a function of the concentration of the external equilibrium solution (*c*): 1—MK-40 in the NH_4_NO_3_ solution, 2—MK-41 in the KNO_3_ solution, 3—MK-41 in the NH_4_NO_3_ solution, 4—MK-40 in the KNO_3_ solution.

To confirm that the sulphonic cation exchange membrane phase is enriched with hydrogen ions in ammonium salts solutions, we can analyze the concentration dependence of the electrical conductivity of membranes with different functional groups in solutions of ammonium and potassium salts. The electrical conductivity of the MK-40 membrane, represented as a function of the concentration of equilibrium solutions NH_4_NO_3_ and KNO_3_, is demonstrated in Figure 7. According to the microheterogenous model of the ion transfer in the membranes [45], it equals the value of electrical conductivity of the “gel phase” of the composite sample, which is a set of charged areas of the membrane’s matrix, whose micropores contain fixed and mobile ions. It is assumed that the electrical conductivity of the gel phase does not depend on the concentration of the solution. Inter-gel areas are filled with an electrically neutral solution (“inter-gel phase”) whose electrical conductivity is proportional to the concentration of the external equilibrium solution. When its concentration lowers, the electrical conductivity of the membrane also decreases.

Any decrease on the electrical conductivity of the membrane and dilution of the equilibrium solution of salts that do not undergo hydrolysis (for instance, KNO_3_) is accounted for by a decrease in the electrical conductivity of the solution in inter-gel regions. Apparently, in the case of NH_4_^+^, the degree and nature of the concentration dependence of the electrical conductivity of the membrane is affected both by a decrease in the conductivity of the inter-gel phase of the membrane and the hydrolysis of the membrane:

R-SO_3_NH_4_ + H_2_O = R-SO_3_H + NH_3_⋅H_2_O, (9)

NH_4_NO_3_ + H_2_O = HNO_3_ + NH_3_⋅H_2_O. (10)

This results in a decrease in the pH of the internal solution of the membrane and the formation of more mobile hydrogen ions.

The same conclusion can be made based on the analysis of the electrical conductivity of the MK-41 phosphonic acid membrane in the form of ammonium cations. We observed a more rapid decrease in electrical conductivity at lower concentrations of the solution. It is likely that, when the external solution is diluted, the hydrogen ions formed as a result of hydrolysis form a weak group:

R-PO_3_(NH_4_)_2_ + H_2_O = R-PO_3_HNH_4_ + NH_3_⋅H_2_O, (11)

R-PO_3_HNH_4_ + H_2_O = R-PO_3_H_2_ + NH_3_⋅H_2_O. (12)

As a result, they do not have any significant influence on the electrical conductivity of the membrane, since in hydrogen form it is lower than in the forms of other single-charged ions.

Earlier studies of the electrical conductivity [46,47] and current-voltage characteristics [48] of anion exchange membranes in solutions of salts of weak inorganic acids also demonstrated that they behave differently from the membranes in strong electrolyte solutions. This is explained by the growth of the pH and enrichment of the internal solution of the membrane with multicharge ions due to the Donnan exclusion of H^+^ ions from the membranes. The cation exchange membrane contacting with the cations of a weak base behaves symmetrically: the concentration of OH^−^ ions is lower than in the solution because they serve as co-ions for this ion exchanger. As a result, the pH of the solution in the membrane decreases as compared to the equilibrium solution. This is also confirmed by the analysis of ionic equilibria in the system cation exchange membrane/ammonium nitrate solution for various concentrations of the salt solution (*c*_p_). A similar calculation was performed in [49] for an anion exchange membrane. The pH of a salt solution which has undergone cationic hydrolysis is determined by the ratio:

(13) $$pH=7-\frac{pK_{d}+lgc_{p}}{2}.$$

Then, the conditions of the electrical neutrality of the external solution (4) and the membrane (5) are presented as follows:

(14) $$c_{NH_{4}^{+}}+c_{H_{}^{+}}=c_{NO_{3}^{-}},$$

(15) $${\bar{c}}_{NH_{4}^{+}}+{\bar{c}}_{H_{}^{+}}=\bar{Q}+{\bar{c}}_{NO_{3}^{-}}.$$

Knowing the ratio of $c_{H^{+}}/c_{NH_{4}{}^{+}}$ in the solution and using the Nikolsky equation (3), we can obtain ${\bar{c}}_{H^{+}}/{\bar{c}}_{NH_{4}{}^{+}}$ for the membrane. Assuming that the membrane is conventionally homogeneous, the concentrations of cations can be expressed per unit of the membrane volume. Another method of calculation assumes a more heterogeneous nature of the ion exchange membrane. Then, the concentrations of cations in the internal solution are calculated in moles per unit of the volume of the internal solution. In this case, the volume fraction of the solution approximates the concentration of water in the membrane (in cm^3^ H_2_O/cm^3^ of the membrane in the swollen state) [36].

**Figure 8:**
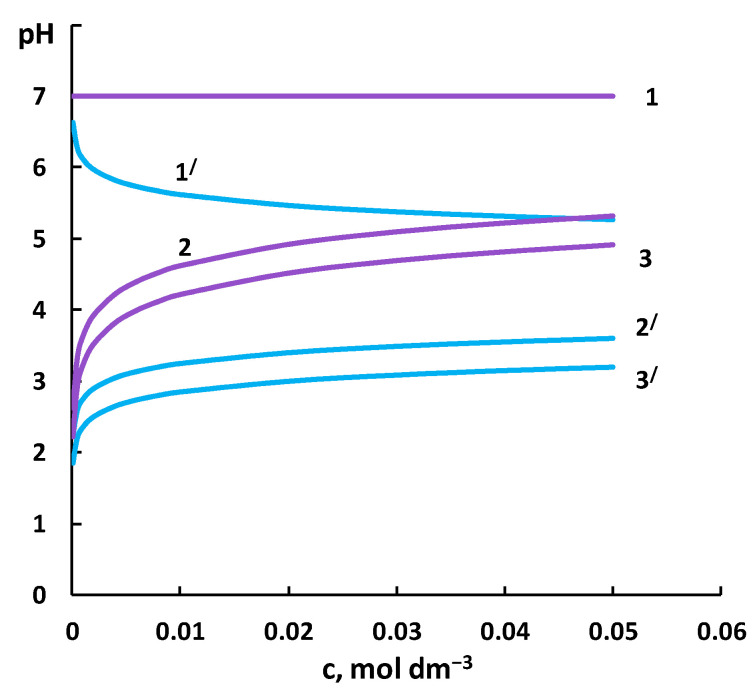
Changes in the pH in the systems “cation exchange membrane/ammonium nitrate solution” (1,2,3) and “cation exchange membrane/potassium nitrate solution” (1′,2′,3′): 1,1′—pH of the external solution; 2,2′—pH inside the membrane calculated without taking into account the heterogeneity of the membrane; 3,3′—pH of the internal solution.

Figure 8 presents the calculated values of the pH depending on the concentrations of NH_4_NO_3_ and KNO_3_. In both cases, the pH inside the cation exchange membrane (pH_internal_) is lower than the pH of the contacting solution (pH_external_). However, the effect is stronger for the ammonium counterion. When the external solution is diluted, the difference ΔpH = pH_external_ – pH_internal_ increases, which agrees with the assumptions suggested above. Taking into account the heterogeneous nature of the membrane, we obtained a lower pH of the internal solution for both salts.

Determining the pH of the internal solution of the membrane is a rather difficult experimental problem. Early studies focusing on the problem [36,37] demonstrated that for anion exchange membranes the difference ΔpH = pH_external_ – pH_internal_ does not equal zero and increases when the external solution is diluted. The effect is stronger for the membrane in the form of anions of a weak acid. The problem was further investigated in [50], where the pH of the solution inside ion exchangers was determined using a mixture of anthocyanins.

An analysis of the system performed using the methodology described in [36] demonstrated that when the MK-40 membrane in the NH_4_^+^ form is put into water, the pH of the external solution increases up to 7.27. Having measured the concentration of NH_4_^+^ in the solution, we used the Nikolsky Equation (3) to determine the pH of the internal solution of the membrane, which was 1.7. In a similar experiment with the MK-40 membrane in the K^+^ form, the pH of the internal solution of the membrane was 4.6.

**Figure 9:**
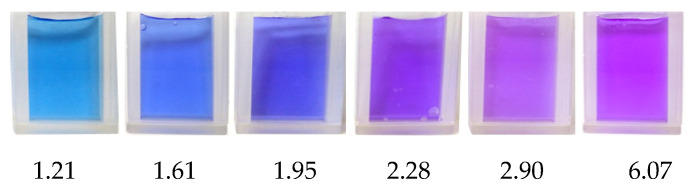
Color of the methyl violet indicator depending on the pH of the solution.

**Figure 10:**
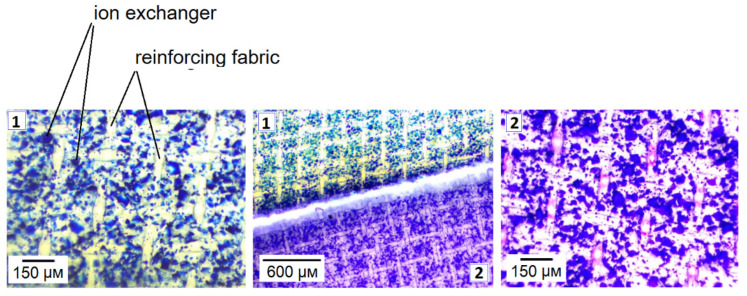
Photographs of sulphonic cation exchange membranes in NH_4_^+^ (1) and K^+^ (2) forms after their contact with a solution containing methyl violet.

**Figure 11:**
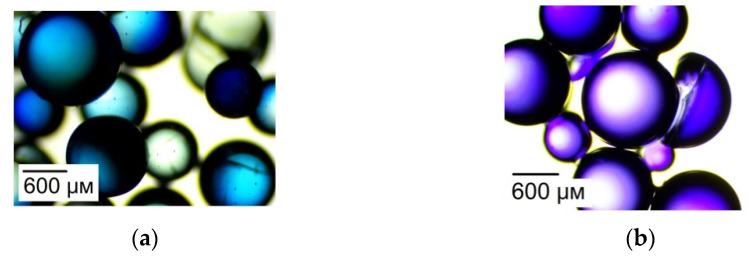
Photographs of KU-2-8 granules in NH_4_^+^ (**a**) and K^+^ (**b**) forms after their contact with a solution containing methyl violet.

Figure 9 demonstrates the color of the methyl violet indicator, when the pH is within the range from 1.21 to 6.07. Micrographs of the membranes and their granular analogues are presented in Figure 10 and Figure 11.

Comparing the color of the KU-2-8 granules and the particles of the ion exchanger in MK-40 to the reference color scale of methyl violet obtained in the experiments (Figure 9), we can say that for the samples in the NH_4_^+^ form, the pH of the internal solution is lower than the pH of the K^+^ form of ion exchangers and does not exceed 2.28. The results are in good agreement with the experimentally calculated values of the pH described above.

To develop theoretical substantiation for the suggested mechanism of electromass transfer and explain the changes in the concentration during electrodialysis of ammonium nitrate, we modelled the electromembrane ion transport in the studied system, taking into account the hydrolysis of the electrolyte in the solution and in the ion exchange membrane. Following the continuum approach [51,52], we considered the membrane as a homogeneous phase—an aqueous solution of the polymer and fixed and mobile ions. Let us assume that the transport of ions in the system is performed by diffusion or migration mechanisms only. Then, describing the one-dimensional electrodiffusion mass transfer (along the *x* axis), flux *J*_i_ of mobile particles of the i-th type (i = K^+^ in KNO_3_ solution or NH_4_^+^ in NH_4_NO_3_ solution, NO_3_^−^, H^+^, OH^−^, NH_3_) can be determined using the Nernst–Planck equation:

(16) $$J_{i}=-D_{i}\left(\frac{dc_{i}}{dx}+z_{i}c_{i}\frac{F}{RT}\frac{d\varphi }{dx}\right).$$

Here *F* is the Faraday constant, *R* is the universal gas constant, *T* is temperature, and φ is the electric potential.

We took into account the fact that, during steady elecrodialysis, the divergence is zero,

(17) $$\nabla J_{i}=0$$

and the Donnan equilibrium is observed on both phase boundaries between the membrane and the external solution (19, 5) [53].

We also considered the possibility of disturbance of the local electrical neutrality in the space charge region near the depleted solution/membrane interface [54,55]. We took into account the fact that when ion exchange membrane was used, mobile ions along with fixed ions (counterions and co-ions) contribute to the total space charge. Nonlinear effects of distribution of the space charge were considered in joint solutions to the Nernst–Planck equation and Poisson’s equation:

(18) $$\varepsilon_{0}\varepsilon_{r}\Delta \varphi =-F\sum_{i}z_{i}c_{i}.$$

Here ε_0_ is the electric constant and ε_r_ is the dielectric permeability, which was assumed to be 80 for the aqueous solution and 30 for the membrane [34].

The equation system (16)–(18) was solved numerically by the finite element method using the COMSOL Multiphysics software package for a one-dimensional three-layer model, which simulated the membrane together with the adjacent interface diffusion layers (Figure 4). An advantage of three-layer models is the possibility of describing the electromass transfer, taking into account the concentration polarization, which results in varied concentrations near phase boundaries, occurrence of the limited state in the desalination compartment [56], and consequently a change in the ion flow.

The initial concentrations of the electrolyte ions were assumed to be equal to the volume concentration of the salt (0.01 M). The concentrations of the components on the outer borders of the studied three-layer model were assumed to be same in the volume of the intermembrane space (0.01 M). The 0.5 mm thick ion exchange membrane was considered as a homogeneous phase, with the concentration of ionogenic groups being 2 M. The thickness of the diffusion layers adjacent to the membrane was assumed to be 0.1 mm.

The reactions of water dissociation and weak base cation hydrolysis in external diffusion layers near the boundary and the internal solution were also considered, assuming that the local chemical equilibrium (1) existed.

When modelling the electrodialysis process, it is commonly assumed that the diffusion coefficients of ions inside the ion exchange membrane are lower than in the aqueous solution. This is due to the movement of ions in an inhomogeneous medium, as well as in the field of action of fixed ions, whose charge density is higher than in the solution. The effect is observed experimentally [57] and is described as the effect of tortuosity and electrostatic interaction [58]. Lower values of ion mobility in the membrane compared to the solution are also confirmed by theoretical calculation, using the example of proton diffusion coefficients in hydrated membranes [59].

Thus, the calculations presented in [60] used diffusion coefficients of ions equal to 10% of their value in the aqueous solution. The diffusion coefficient of the proton was assumed to be 60% of its value in an aqueous medium. In [34], the diffusion coefficients of ions in the membrane were 10^−11^ m^2^/s, and the diffusion coefficients of OH^−^ and H^+^ were quite high, only three times lower than the corresponding values in the solution. This allowed the authors to obtain good agreement between the experimental and theoretical dependences of the diffusion permeability of the anion exchange membrane.

In this paper, we found the diffusion coefficients for all ions in the membrane from the electrical conductivity of the membrane. We proposed a uniform approach for all counterions. We measured the electrical conductivity of an ion-exchange membrane in different ionic forms and calculated the diffusion coefficients of counterions in the membrane, using the Nernst–Einstein equation at the maximum dilution of the external solution:

(19) $${\bar{D}}_{i}={\bar{\lambda }}_{i}\frac{RT}{z_{i}^{2}F^{2}}.$$

Here, ${\bar{\lambda }}_{i}$ is the mobility of the ion in the membrane calculated based on the molar electrical conductivity $\bar{\Lambda }$ of the membrane

(20) $$\bar{\Lambda }=\frac{k_{m}}{\bar{c}};\;\bar{\Lambda }=\sum_{}^{}\nu_{i}{\bar{\lambda }}_{i}.$$

Diffusion coefficients of the ions in the membrane and other parameters of the modelling are given in Table 3. For aqueous solutions, diffusion coefficients of ions and molecules presented in Table 1 were used.

The distribution of the concentrations of the components and pH in the membrane and the diffusion layers, as well as the component fluxes, were calculated for potentiostatic conditions of electrodialysis with a constant difference in the potentials on the outer boundaries of the considered one-dimensional model.

**Figure 12:**
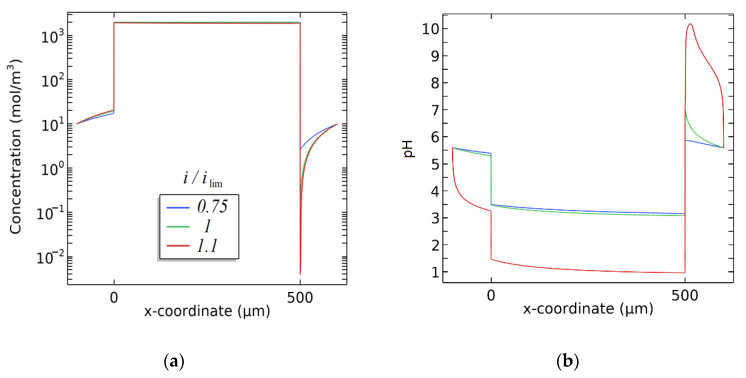
Numerical calculation of the distribution of the concentration of cation (**a**) and the pH (**b**) during electrodialysis of the NH_4_NO_3_ solution.

**Figure 13:**
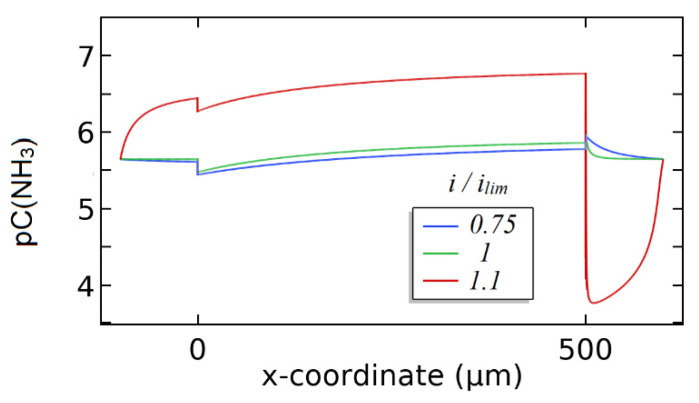
Numerical calculations of the distribution of ammonia during electrodialysis of the NH_4_NO_3_ solution.

The results are presented in Figure 12 and Figure 13. We can see that the concentration of the counterion (NH_4_^+^) inside the membrane equals the ion exchange capacity of the membrane (Figure 12a), which can be explained by the Donnan equilibrium [60]. Near the membrane, the concentration of the counterion decreases in the depleted layer and increases in the enriched layer because of the concentration polarization. The gradient of electrolyte cation concentration in the depleted solution increases with the growth of the current density.

The calculation of the changes in the pH in the studied system are demonstrated in Figure 12b. When the current does not exceed the limiting diffusion value, the pH of the depleted solution near the membrane is determined by means of hydrolysis of the salt of the weak base and equals 5.6. When *i = i*_lim_, the local pH of the depleted solution grows up to 7, presumably due to the beginning of the decomposition of the water molecules on the membrane/solution interface and migration of hydrogen ions through the cation exchange membrane.

When the current density slightly exceeds the limiting value (*i/i*_lim_ = 1.1), the pH of the depleted solution on the boundary with the membrane grows rapidly to 10. While at *i/i*_lim_ = 0.75–1 the solution in the concentrate compartment is slightly acidified, at *i/i*_lim_ = 1.1 its pH decreases rapidly to 3.2. The pH inside the membrane also decreases from 3.3 to 1.0 when the current density slightly exceeds the limiting current. We should note that in this case the membrane is presented as a homogeneous sample and the measured pH is not the pH of the internal solution, but rather a mean pH calculated for the whole volume of the ion exchanger. The rapid decrease in the pH is most likely accounted for by the beginning of active dissociation of water on the depleted membrane/solution interface.

Figure 13 shows numerical calculations of the distribution of the concentration of NH_3_ during electrodialysis of the ammonium nitrate solution. The shift of the pH on the depleted membrane/solution interface results in a rapid growth in the concentration of NH_3_ near the surface of the membrane, namely by about two orders of magnitude (pC decreases from 5.5 to 3.8). The concentration of NH_3_ inside the membrane drops because of acidification. Acidification of the solution in the concentrate compartment also leads to a decrease in the concentration of NH_3_ in the enriched diffusion boundary layer. The concentration of NH_3_ in the system depends on the pH in each of the three studied phases.

**Figure 14:**
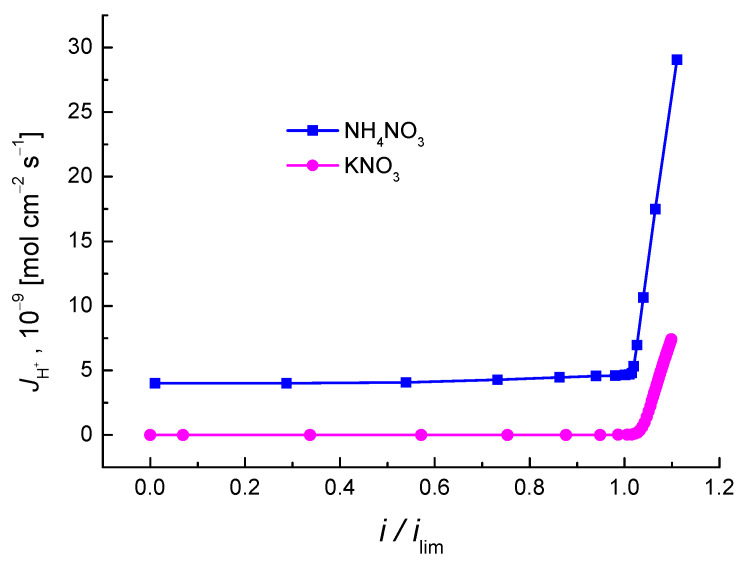
Dependence of the flux of hydrogen ions on the dimensionless current density.

Analysis of the calculations of the average hydrogen ions flux in the concentrate compartment (Figure 14) confirms the results of the experiment (Figure 5). Indeed, if the salt undergoes hydrolysis in an aqueous solution with under-limiting current densities, the flux of hydrogen ions is negligibly small and grows significantly when the current density exceeds *i*_lim_. At the same time, if the salt is formed by a weak base, the flux of hydrogen ions is relatively large, irrespective of whether the limiting current density is reached or not. This can be explained by the acidification of the salt solution as a result of hydrolysis (1). When the limiting current is exceeded, the decrease in the pH results in further growth of the flow of hydrogen ions.

## 4. Conclusions

The paper used an approach designed for the description of the transport of anions of the salts of weak acids, taking into account the hydrolysis reaction in the external diffusion boundary layer and the internal solution of the membrane. Based on this approach, we developed a mechanism of electromass transfer of cation of a weak base (using the case of ammonium ions) through the cation exchange membrane. The study demonstrated that, at under-limiting currents, the fluxes of hydrogen ions through the membrane are negligibly small for potassium nitrate, which is not the same for ammonium nitrate, which undergoes hydrolysis.

Our experiments and mathematical simulation of the diffusion and migration transfer of components of the studied electromembrane system confirmed that the pH of the ammonium nitrate solution changes both in the near-membrane diffusion layers and in the membrane phase. The acidity of the depleted solution decreases, while the acidity of the enriched solution, on the contrary, increases, which can be considered as an additional transfer of H^+^ ions through the cation exchange membrane. Due to the lower pH of the internal membrane solution, NH_3_ transforms into NH_4_^+^ inside the cation exchanger. Removal of the hydroxyl ions to the depleted layer results in the acidification of the solution near the membrane in the concentrate compartment. Ammonium migrates to the enriched solution, where it interacts with water molecules, which results in the acidification of the solution in the concentrate compartment.

The obtained results are of great practical significance. They indicate that during the electrodialysis of a wastewater solution containing ammonium and calcium, magnesium, or iron cations (for instance, by-products of manufacturing mineral fertilizers), even at under-limiting currents, the increased pH may lead to the formation of poorly soluble hydroxides not only in the anion exchange membranes in the concentrate compartment, but on the receiving side of the cation exchange membrane in the desalination compartment as well.

## Figures and Tables

**Table 1:** Characteristics of heterogeneous ion-exchange membranes [38].

Membrane	Functional Groups	Binder	Reinforcing Fabric	Thickness, mm	Exchange Capacity, mmol g^−1^	Moisture Contents, %	Transport Number	Electrical Resistivity (in 0.6 M NaCl) Ohm⸱cm
MK-40	-SO_3_H	Polyethylene	Capron	0.41 ± 0.04	2.40 ± 0.22	40 ± 5	>0.80	220
MK-41	-PO_3_H_2_	Polyethylene	Capron	0.50 ± 0.04	2.80 ± 0.23	35 ± 4	>0.80	350
MA-41	-N^+^(CH_3_)_3_	Polyethylene	Capron	0.55 ± 0.05	2.00 ± 0.16	40 ± 5	>0.94	350

**Table 2:** Characteristics of the components of the studied solutions [18,39,40].

Particle	Stokes Radius, nm	Diffusion Coefficient in Solution, D_i_·10^9^, m^2^/s
H^+^	0.026	9.311
K^+^	0.125	1.957
NH_4_^+^	0.124	1.957
NO_3_^-^	0.128	1.902
NH_3_	0.130	1.705
OH^–^	0.046	5.273

**Table 3:** Simulation parameters.

Parameter	Value and Measurement Unit
Thickness of the diffusion layer	100 μm
Thickness of the membrane	500 μm
Total exchange capacity of the membrane	2 mole/L
Diffusion coefficient of H^+^ in the membrane	1.4 × 10^−10^ m^2^ s^−1^
Diffusion coefficient of OH^–^ in the membrane	0.8 × 10^−10^ m^2^ s^−1^
Diffusion coefficient of NH_4_^+^ in the membrane	5.4 × 10^−11^ m^2^ s^−1^
Diffusion coefficient of K^+^ in the membrane	5.4 × 10^−11^ m^2^ s^−1^
Diffusion coefficient of NO_3_^−^ in the membrane	5.4 × 10^−11^ m^2^ s^−1^
Diffusion coefficient of NH_3_ in the membrane	4.74 × 10^−11^ m^2^ s^−1^
